# Supplementary material for: Increasing temperatures affect thoracic muscle performance in Arctic bumblebees
Source: Nat Commun. 2025 Nov 3;16:9699. doi: 10.1038/s41467-025-65671-6 (PMC12583728; doi:10.1038/s41467-025-65671-6)
Supplement: Supplementary file 1 — Supplementary Information [file 41467_2025_65671_MOESM1_ESM.pdf]

Supplemental Material For:

Increasing temperatures affect thoracic muscle performance in Arctic bumblebees

Charlie Woodrow[1*†], Guadalupe Sepúlveda-Rodríguez[2†], Samyuktha Rajan[2], Michael Mitschke[2,3], Emily Baird[2‡], Mario Vallejo-Marín[1‡]

[1]Department of Ecology and Genetics, Uppsala University, Evolutionary Biology Centre, Norbyvägen 18 D, 752 36, Uppsala, Sweden

[2]Department of Zoology, Stockholm University, Svante Arrhenius väg 18 B, 114 18, Stockholm, Sweden

[3]Centre for Palaeogenetics, Stockholm University, Svante Arrhenius väg 20 C, 114 18, Stockholm, Sweden

[*]Correspondence: charlie.woodrow@ebc.uu.se

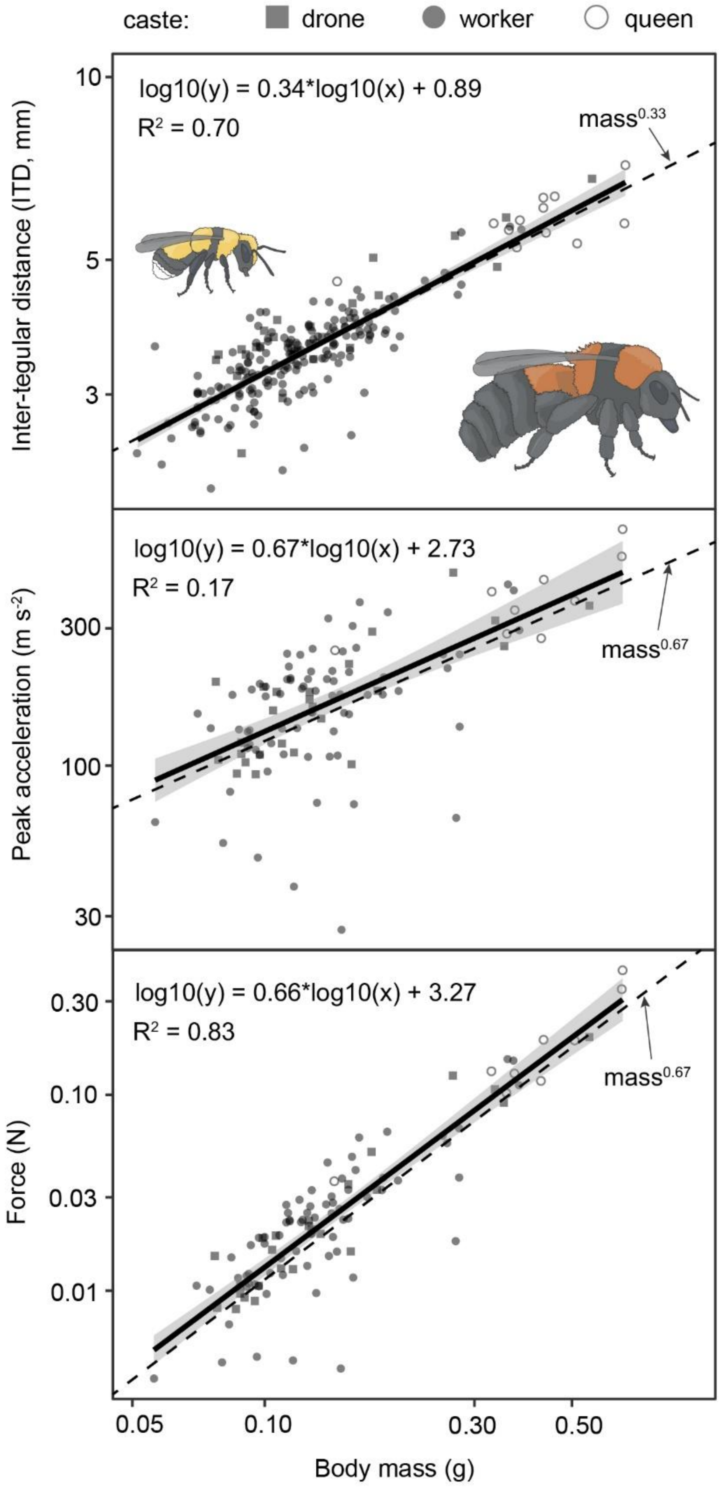

Fig. S1: Isometric scaling of bee size and thorax vibration amplitude in Arctic bumblebees. Scaling relationships between mass and inter-tegular distance (ITD, top), peak acceleration (middle), and force (bottom). Under isometric scaling, ITD should scale with the same exponent as a mass and length relationship of $mass^{0.33}$, while peak acceleration and force should scale with the same exponent of a mass and cross-sectional area (CSA) relationship ($mass^{0.67}$), as force is linearly correlated with muscle CSA. Dashed lines indicate the relationships expected under isometry. Solid lines indicates model fits with 95% CIs.

Table S1. Summary statistics of Arctic bumblebee (*Bombus* spp.) distribution classes, size measurements, and defensive buzz properties for individuals measured in the field. All data were averaged for all observations in each species and caste combination. N = number of individuals, n = number of buzzes. All values represent means ± standard deviations. For queens captured at sites 3, 4 and 5 (see Fig. 1a) we were unable to record mass (N = 6). ITD = Inter-tegular distance.

Species	Distribution class	Caste	N	n	ITD (mm)	Mass	Peak acceleration ($m\ s^{-2}$)	Fundamental frequency (Hz)	Duration (s)
B. alpinus	specialist	drone	3	107	4.54 ± 0.96	0.09 ± 0.03	206.86 ± 96.14	216.55 ± 68.42	0.47 ± 0.49
		queen	2	15	6.25 ± 0.13	n/a	375.38 ± 185.75	190.29 ± 67.56	0.41 ± 0.45
		worker	5	256	4.38 ± 0.79	0.18 ± 0.07	248.39 ± 138.12	260.02 ± 61.78	0.32 ± 0.33
B. balteatus	specialist	drone	2	117	5.15 ± 0.27	0.33 ± 0.01	359.04 ± 187.64	218.09 ± 43.53	0.45 ± 0.45
		queen	4	140	6.87 ± 0.31	0.65 ± 0.01	472.07 ± 290.96	171.05 ± 45.95	0.84 ± 1.19
		worker	3	189	4.37 ± 0.07	0.17 ± 0.01	318.42 ± 131.30	236.11 ± 59.36	0.45 ± 0.41
B. bohemicus	generalist	drone	1	48	5.04	0.18	292.10 ± 119.38	192.43 ± 42.32	0.54 ± 0.60
B. cingulatus	generalist	drone	1	53	2.40	0.09	120.33 ± 71.04	170.23 ± 27.37	0.81 ± 1.36
B. flavidus	generalist	drone	3	220	3.59 ± 0.12	0.12 ± 0.02	136.99 ± 73.82	188.78 ± 44.11	0.41 ± 0.39
B. hortorum	generalist	queen	1	62	5.74	0.33	402.00 ± 159.56	205.39 ± 46.76	0.30 ± 0.19
		worker	4	174	4.77 ± 0.83	0.28 ± 0.07	252.38 ± 199.48	213.12 ± 62.63	0.70 ± 1.16
B. hyperboreus	specialist	drone	3	149	6.12 ± 0.57	0.40 ± 0.12	364.29 ± 179.18	243.95 ± 62.53	0.56 ± 0.76
		queen	3	233	9.20 ± 1.07	n/a	269.65 ± 182.77	262.21 ± 46.48	0.53 ± 0.54
B. jonellus	generalist	drone	2	105	3.58 ± 0.01	0.09 ± 0.01	99.19 ± 53.85	226.83 ± 42.77	0.58 ± 0.83

		queen	2	59	5.48 ± 0.12	0.42 ± 0.02	423.21 ± 241.25	179.59 ± 41.39	0.71 ± 0.70
		worker	14	805	3.51 ± 0.29	0.12 ± 0.04	153.43 ± 104.86	229.20 ± 69.18	0.43 ± 0.54
B. lapponicus	specialist	drone	2	122	3.59 ± 0.07	0.11 ± 0.02	153.81 ± 74.71	273.53 ± 79.44	0.25 ± 0.15
		queen	1	60	5.32	0.51	373.47 ± 129.34	265.18 ± 51.41	0.16 ± 0.07
		worker	9	562	3.69 ± 0.36	0.11 ± 0.01	166.74 ± 90.75	206.86 ± 57.67	0.46 ± 0.56
B. lucorum	generalist	drone	2	88	3.85 ± 0.28	0.16 ± 0.01	187.18 ± 107.29	226.98 ± 56.49	0.76 ± 1.31
		worker	13	576	3.64 ± 0.15	0.14 ± 0.02	166.99 ± 96.55	199.72 ± 51.33	0.57 ± 0.75
B. monticola	generalist	drone	7	412	3.68 ± 0.25	0.10 ± 0.01	146.09 ± 78.27	213.13 ± 43.38	0.38 ± 0.38
		queen	1	68	5.81	n/a	244.31 ± 126.81	199.44 ± 52.66	0.18 ± 0.13
		worker	3	142	2.78 ± 0.65	0.08 ± 0.02	162.93 ± 85.46	201.45 ± 51.77	0.59 ± 0.72
B. pascuorum	generalist	drone	1	50	2.96	0.11	119.30 ± 75.51	172.62 ± 24.53	0.48 ± 0.60
		queen	3	190	5.84 ± 0.30	0.47 ± 0.13	364.54 ± 205.05	186.18 ± 55.62	0.45 ± 0.60
		worker	10	454	3.87 ± 0.45	0.16 ± 0.06	155.20 ± 111.31	212.78 ± 61.42	0.53 ± 0.92
B. pyrrhopygus (synonym polaris)	specialist	queen	1	28	4.21	0.14	251.35 ± 148.31	186.92 ± 37.13	0.32 ± 0.15
		worker	1	71	5.35	0.37	406.15 ± 185.54	232.00 ± 54.84	0.28 ± 0.18
B. pratorum	generalist	worker	9	552	3.57 ± 0.44	0.10 ± 0.02	149.43 ± 86.06	208.98 ± 72.57	0.44 ± 0.50
B. mastrucatus (synonym wurflenii)	generalist	drone	1	26	4.37	0.18	181.84 ± 78.55	269.54 ± 64.17	0.17 ± 0.10
		worker	1	63	4.09	0.16	305.60 ± 103.86	196.47 ± 39.47	0.47 ± 0.39

Table S2. Summary of linear model (one-sided) for log-transformed mass and acceleration. The previous model (Table S5) found that caste had no effect, so was removed. Data was log transformed to compare the estimate for mass to the slope expected under isometry (log(acceleration) ~ log(body mass) $^{0.67}$). Data were analysed as model summaries. Observations are averaged per bee (N = 118 bees of 15 species). P-value not adjusted.

Model	Estimate	Std. Error	F-value	*P*-value
log10(acceleration) ~ log10(mass)				
Intercept	2.74	0.02		
Log10(mass)	0.67	0.02	1134.3	2.2e-16 ***

Table S3. Location and description of field sites. Site names, GPS, elevation and general observational descriptions.

Site name	N	E	Elevation	Habitat type	Observations
Bridge ditch	68.352	18.815	~410m	Wayside	*Epilobium angustifolium, Trifolium repens, Trifolium pratense*
Mossy Lake	68.330	18.832	~500m	Bog	Few flowers, yellow Asteraceae (*Solidago virgaurea*?)
Nuolja	68.372	18.695	~1100m	Alpine	*Vaccinium vitis*
Björkliden Skua	68.411	18.611	~800m	Subalpine	Diverse, most bees on blue Fabaceae
Trollsjön	68.384	18.345	~815m	Subalpine	Very diverse subalpine meadow

Table S4. Exceptions for extracting buzzes from accelerometer recordings. The default settings for detecting a buzz within the 60 second recordings included a minimum threshold of 8 % of the peak amplitude, and a minimum duration of 0.1 seconds. For some recordings however, these defaults were changed.

Recording	Change in analysis	Reasoning
B_alpinus_queen_queens_141151.txt	used threshold of 2%	a few high amplitude spike artifacts
B_alpinus_worker_field_121042.txt	used threshold of 4%	a few high amplitude spike artifacts
B_lucorum_worker_field_132258.txt	used threshold of 4%	small buzzes being missed/grouped
B_pascuorum_queen_queens_160340.txt	used threshold of 5%	small buzzes being missed/grouped

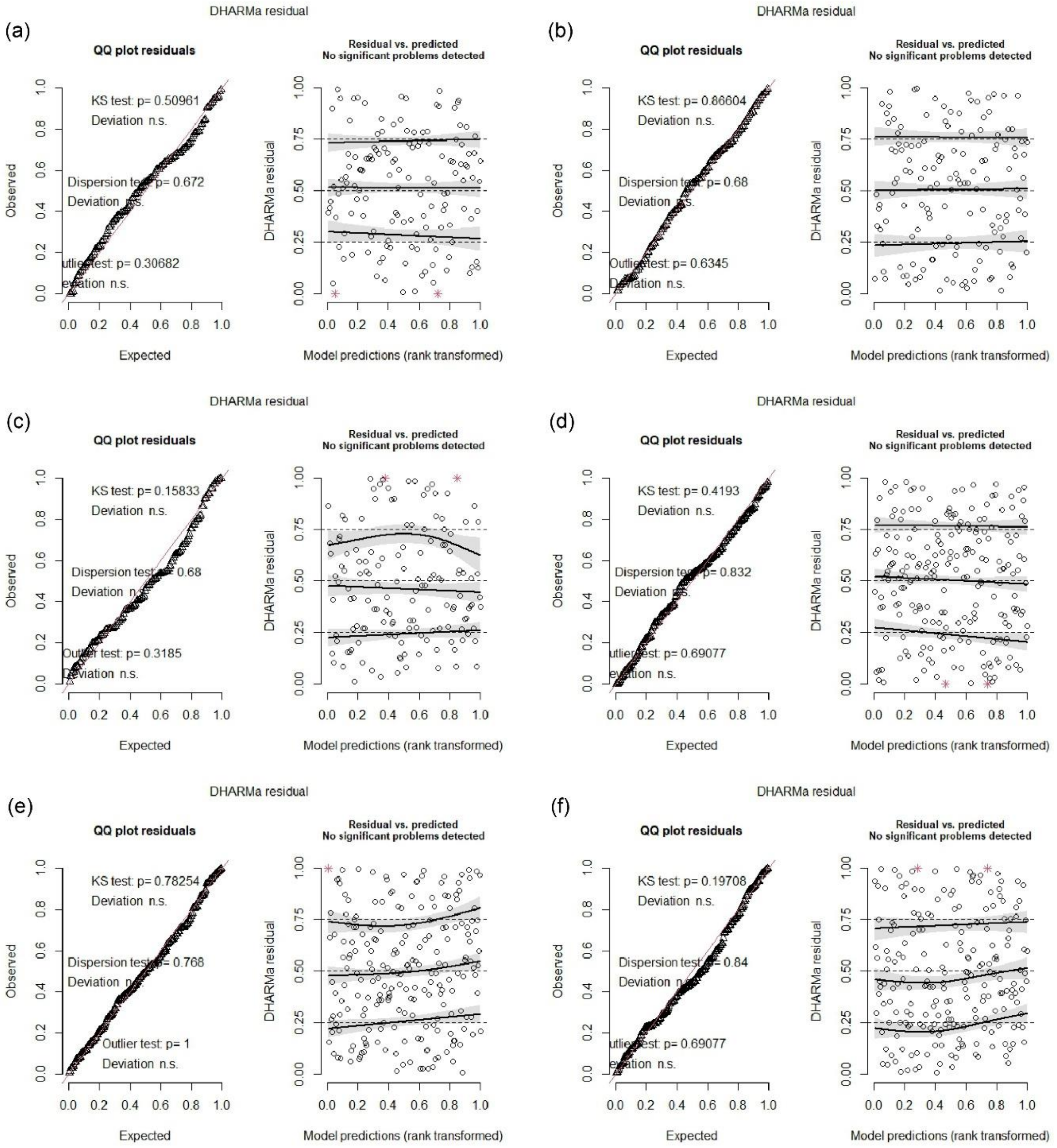

Fig. S2: QQ plots and residual vs model prediction plots using DHARMa[1] to assess the assumptions of the models used in Fig. 2. The model used is considered appropriate for the data if the left panel shows a uniform distribution and the right panel shows no patterns between residuals and model predictions. Models: (a) peak acceleration ~ mass + species + caste. (b) fundamental frequency ~ mass + species + caste. (c) buzz duration ~ mass + species + caste. (d) peak acceleration ~ mass + distribution + (1|species) + (1|caste). (e) fundamental frequency ~ mass + distribution + (1|species) + (1|caste). (f) buzz duration ~ mass + distribution + (1|species) + (1|caste).

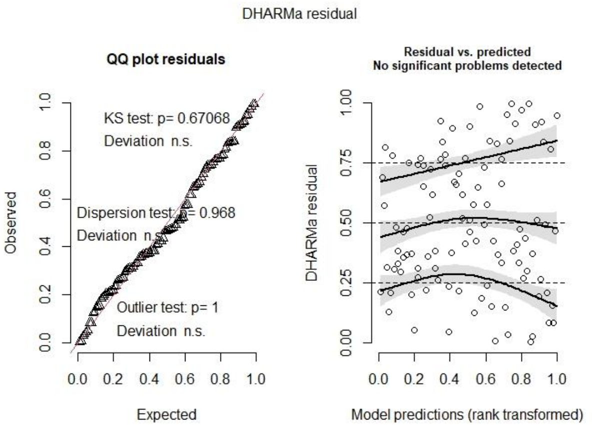

Fig. S3: QQ plots and residual vs model prediction plots using DHARMa[1] to assess the assumptions of the model testing for isometry of the thoracic muscles. Model = log10(peak acceleration) ~ log10(mass).

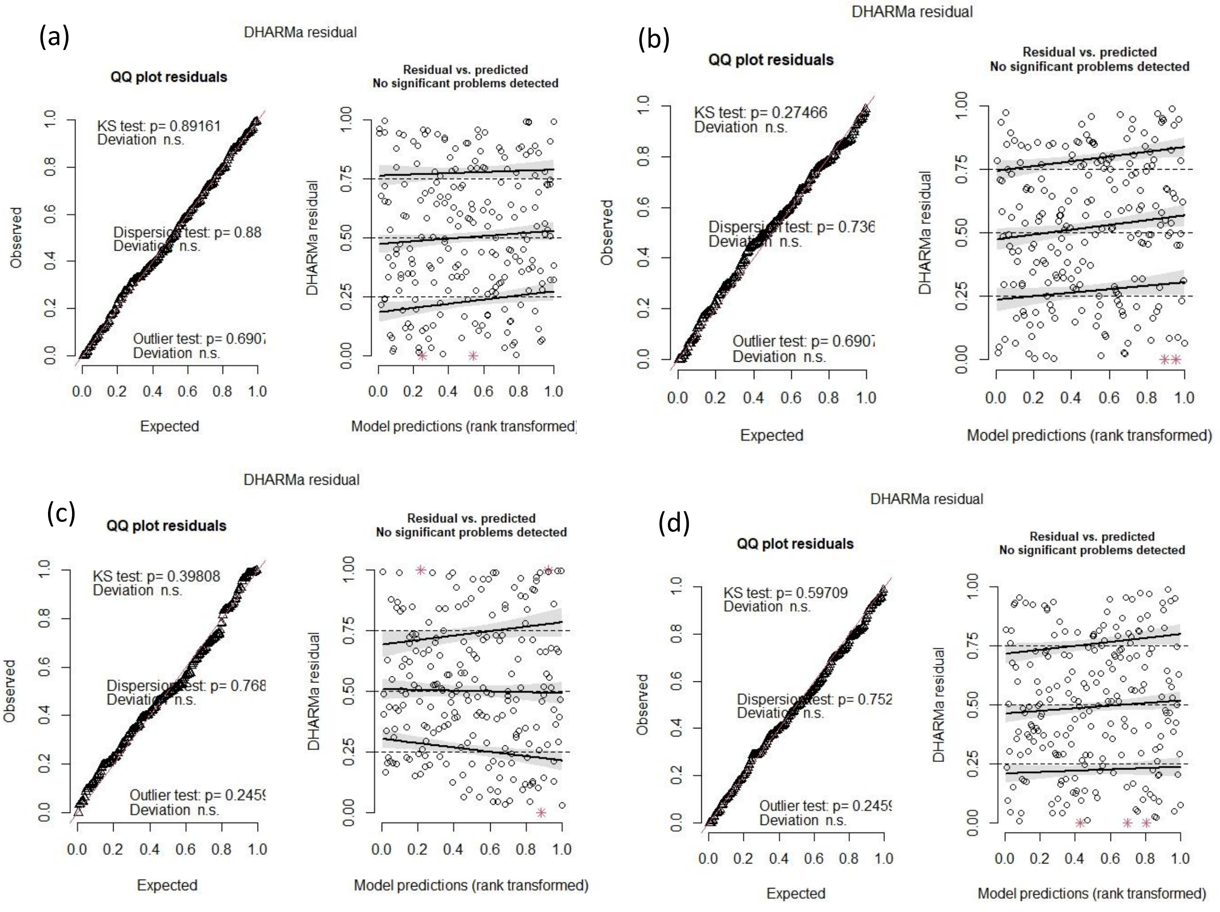

Fig. S4: QQ plots and residual vs model prediction plots using DHARMa[1] to assess the assumptions of the models used in Fig. 3. Models: (a) fundamental frequency ~ air temp + distribution + (1|species) + (1|caste). (b) peak acceleration ~ air temp + distribution + (1|species) + (1|caste). (c) fundamental frequency ~ thorax temp + distribution + (1|species) + (1|caste). (d) peak acceleration ~ thorax temp + distribution + (1|species) + (1|caste).

Table S5. Effect sizes for statistical models behind the analysis in main text Figure 3 and Table 2. We calculated Cohen's f^2 and Cohen's d estimates for effect sizes. Interpretations follow the conventions for both statistics (Cohen's f^2: $f^2 > 0.02$ = small effect, $f^2 > 0.15$ = medium effect, $f^2 > 0.35$ = large effect; Cohen's d: d > 0.2 = small effect, d > 0.5 = medium effect, d > 0.8 = large effect). NS = term not significant.

	Model	Term	t Value	df	F Value	f^2	d	Interpretation
(a)	Frequency ~ T_{air} + distribution + (1 | species) + (1 | caste)	T_{air}	3.998	210.63	15.99	0.076	0.275	Small-Medium effect
(b)	Acceleration ~ T_{air} + T_{air}^2 + Mass + distribution + (1|species) + (1 | caste)	T_{air}	4.029	201.85	16.23	0.080	0.283	Small-Medium effect
		T_{air}^2	-3.738	203.12	13.97	0.069	0.262	Small-Medium effect
		Mass	8.361	75.16	69.91	0.921	0.964	Large effect
(c)	Frequency ~ T_{thorax} + T_{thorax}^2 + distribution + (1|species) + (1 | caste)	T_{air}	-1.550	205.09	2.40	0.012	0.108	Small (NS)
		T_{air}^2	3.125	204.01	9.76	0.048	0.218	Small-Medium effect
(d)	Acceleration ~ T_{thorax} + T_{thorax}^2 + Mass + distribution + (1|species) + (1 | caste)	T_{air}	3.084	209.05	9.51	0.046	0.213	Small-Medium effect
		T_{air}^2	-2.470	208.64	6.10	0.029	0.171	Small effect
		Mass	7.559	95.95	57.14	0.596	0.770	Large effect

References

1. Hartig, F. DHARMa: Residual Diagnostics for Hierarchical (Multi-Level / Mixed) Regression Models. https://CRAN.R-project.org/package=DHARMa (2022).
