# Supplementary material for: Increasing temperatures affect thoracic muscle performance in Arctic bumblebees
Source: Nat Commun. 2025 Nov 3;16:9699. doi: 10.1038/s41467-025-65671-6 (PMC12583728; doi:10.1038/s41467-025-65671-6)
Supplement: Supplementary file 2 — Reporting Summary [file 41467_2025_65671_MOESM2_ESM.pdf]

nature portfolio

Corresponding author(s): Dr Charlie Woodrow

Last updated by author(s): Feb 27, 2025

Reporting Summary

Nature Portfolio wishes to improve the reproducibility of the work that we publish. This form provides structure for consistency and transparency in reporting. For further information on Nature Portfolio policies, see our Editorial Policies and the Editorial Policy Checklist.

Statistics

For all statistical analyses, confirm that the following items are present in the figure legend, table legend, main text, or Methods section.

n/a	Confirmed	
☐	☒	The exact sample size (*n*) for each experimental group/condition, given as a discrete number and unit of measurement
☐	☒	A statement on whether measurements were taken from distinct samples or whether the same sample was measured repeatedly
☐	☒	The statistical test(s) used AND whether they are one- or two-sided<br>*Only common tests should be described solely by name; describe more complex techniques in the Methods section.*
☐	☒	A description of all covariates tested
☐	☒	A description of any assumptions or corrections, such as tests of normality and adjustment for multiple comparisons
☐	☒	A full description of the statistical parameters including central tendency (e.g. means) or other basic estimates (e.g. regression coefficient) AND variation (e.g. standard deviation) or associated estimates of uncertainty (e.g. confidence intervals)
☐	☒	For null hypothesis testing, the test statistic (e.g. *F*, *t*, *r*) with confidence intervals, effect sizes, degrees of freedom and *P* value noted<br>*Give P values as exact values whenever suitable.*
☒	☐	For Bayesian analysis, information on the choice of priors and Markov chain Monte Carlo settings
☐	☒	For hierarchical and complex designs, identification of the appropriate level for tests and full reporting of outcomes
☒	☐	Estimates of effect sizes (e.g. Cohen's *d*, Pearson's *r*), indicating how they were calculated

Our web collection on statistics for biologists contains articles on many of the points above.

Software and code

Policy information about availability of computer code

Data collection	LabView NXG 5.0
Data analysis	R (studio) 4.3.3; seewave (Rpackage); tuneR (Rpackage); lmer (Rpackage); DHARMa (Rpackage); minpack.lm (Rpackage)

For manuscripts utilizing custom algorithms or software that are central to the research but not yet described in published literature, software must be made available to editors and reviewers. We strongly encourage code deposition in a community repository (e.g. GitHub). See the Nature Portfolio guidelines for submitting code & software for further information.

Data

Policy information about availability of data

All manuscripts must include a data availability statement. This statement should provide the following information, where applicable:

- Accession codes, unique identifiers, or web links for publicly available datasets
- A description of any restrictions on data availability
- For clinical datasets or third party data, please ensure that the statement adheres to our policy

The raw buzz data generated in this study have been deposited in Figshare under the accession code XX. Other data, and details of models and assumptions are available in either the main text or supplementary materials.

Research involving human participants, their data, or biological material

Policy information about studies with human participants or human data. See also policy information about sex, gender (identity/presentation), and sexual orientation and race, ethnicity and racism.

Reporting on sex and gender	*Use the terms sex (biological attribute) and gender (shaped by social and cultural circumstances) carefully in order to avoid confusing both terms. Indicate if findings apply to only one sex or gender; describe whether sex and gender were considered in study design; whether sex and/or gender was determined based on self-reporting or assigned and methods used.* *Provide in the source data disaggregated sex and gender data, where this information has been collected, and if consent has been obtained for sharing of individual-level data; provide overall numbers in this Reporting Summary. Please state if this information has not been collected.* *Report sex- and gender-based analyses where performed, justify reasons for lack of sex- and gender-based analysis.*
Reporting on race, ethnicity, or other socially relevant groupings	*Please specify the socially constructed or socially relevant categorization variable(s) used in your manuscript and explain why they were used. Please note that such variables should not be used as proxies for other socially constructed/relevant variables (for example, race or ethnicity should not be used as a proxy for socioeconomic status).* *Provide clear definitions of the relevant terms used, how they were provided (by the participants/respondents, the researchers, or third parties), and the method(s) used to classify people into the different categories (e.g. self-report, census or administrative data, social media data, etc.)* *Please provide details about how you controlled for confounding variables in your analyses.*
Population characteristics	*Describe the covariate-relevant population characteristics of the human research participants (e.g. age, genotypic information, past and current diagnosis and treatment categories). If you filled out the behavioural & social sciences study design questions and have nothing to add here, write "See above."*
Recruitment	*Describe how participants were recruited. Outline any potential self-selection bias or other biases that may be present and how these are likely to impact results.*
Ethics oversight	*Identify the organization(s) that approved the study protocol.*

Note that full information on the approval of the study protocol must also be provided in the manuscript.

Field-specific reporting

Please select the one below that is the best fit for your research. If you are not sure, read the appropriate sections before making your selection.

☐ Life sciences ☐ Behavioural & social sciences ☒ Ecological, evolutionary & environmental sciences

For a reference copy of the document with all sections, see nature.com/documents/nr-reporting-summary-flat.pdf

Ecological, evolutionary & environmental sciences study design

All studies must disclose on these points even when the disclosure is negative.

Study description	We measured the non-flight vibrations of bumblebees using an accelerometer in the lab and field. We used opportunistic sampling to cover a broad species and caste range, with a maximum number of samples to avoid over sampling. We visited multiple field locations to aid in our species coverage.
Research sample	15 species of wild-caught bumblebees (Bombus, Apidae). Each species was sampled for less than 20 individuals, with a sampling strategy in place to avoid over-sampling. This number of samples is representative of only a small proportion of populations.
Sampling strategy	Bees were selected using non-discriminant opportunistic sampling unless a maximum number of individuals per species and caste (4) was reached per site.
Data collection	After capture, bees were held in plastic 15 ml vials that were then placed on ice to reduce activity before handling. Bees were identified to species level using field guides during the time to recover from cooling. Accelerometer measurements were then taken within 1 h of capture. Workers and drones were brought to the laboratory for mass and inter-tegular distance (ITD) measurements and preservation, while queens were released in the field.
Timing and spatial scale	Fieldwork was conducted in Abisko (Ábeskovvu; Lat: 68.35 °N, Long: 18.8 °E) in the Arctic region of Sweden from 12-23 July 2024.
Data exclusions	For some analyses, queens were excluded as we did not measure their thorax temperatures. This is detailed in the manuscript methodology, results, and display items where necessary.
Reproducibility	To maintain reproducibility, we ensured the bees were held in a consistent way and measured for a constant duration. We detail with text and display items the methodology for reproducibility by future studies.
Randomization	Randomization was not entirely necessary for the study as all involved in data collection needed to be confident in the species identification before sampling.
Blinding	Samples were given codes (letter number combinations) so that during data processing there was no way to identify the species or

other variables.

Did the study involve field work? [x] Yes [ ] No

Field work, collection and transport

Field conditions	Temperature conditions are reported in the manuscript as form a key component of the results. Rainfall was minimal during the field season, with high temperatures for the time of year and strong sunlight conditions.
Location	Abisko (Åbeskovvu; Lat: 68.35 °N, Long: 18.8 °E) in the Arctic region of Sweden
Access & import/export	The project was supported by the Bolin Centre for Climate Research. Collection permits are required for collection within the national park regions, so we collected outside of these regions. We made sure to adhere to these collection rules and set criteria for maximum numbers of samples per site and species to prevent oversampling.
Disturbance	We reduced disturbance by sticking to existing footpaths unless absolutely necessary to deviate.

Reporting for specific materials, systems and methods

We require information from authors about some types of materials, experimental systems and methods used in many studies. Here, indicate whether each material, system or method listed is relevant to your study. If you are not sure if a list item applies to your research, read the appropriate section before selecting a response.

Materials & experimental systems

n/a	Involved in the study	
[x]	[ ]	Antibodies
[x]	[ ]	Eukaryotic cell lines
[x]	[ ]	Palaeontology and archaeology
[ ]	[x]	Animals and other organisms
[x]	[ ]	Clinical data
[x]	[ ]	Dual use research of concern
[x]	[ ]	Plants

Methods

n/a	Involved in the study	
[x]	[ ]	ChIP-seq
[x]	[ ]	Flow cytometry
[x]	[ ]	MRI-based neuroimaging

Animals and other research organisms

Policy information about studies involving animals; ARRIVE guidelines recommended for reporting animal research, and Sex and Gender in Research

Laboratory animals	n/a
Wild animals	15 species of wild-caught bumblebees (Bombus, Apidae). Each species was sampled for less than 20 individuals, with a sampling strategy in place to avoid over-sampling. After capture, bees were held in plastic 15 ml vials that were then placed on ice to reduce activity before handling. Bees were identified to species level using field guides during the time to recover from cooling. Accelerometer measurements were then taken within 1 h of capture. Workers and drones were brought to the laboratory for mass and inter-tegular distance (ITD) measurements and preservation in ethanol, while queens were released in the field.
Reporting on sex	Sex-based analyses were conducted as explained in the manuscript. We expected differences in thermal performance between males (drones) and females (workers, queens).
Field-collected samples	Field collected samples were measured in a similar way to wild animals section. Individuals were transported on ice from the field to the laboratory.
Ethics oversight	No ethical guidance is required for the collection of insect samples at the time of sampling.

Note that full information on the approval of the study protocol must also be provided in the manuscript.

Plants

Seed stocks	*Report on the source of all seed stocks or other plant material used. If applicable, state the seed stock centre and catalogue number. If plant specimens were collected from the field, describe the collection location, date and sampling procedures.*
Novel plant genotypes	*Describe the methods by which all novel plant genotypes were produced. This includes those generated by transgenic approaches, gene editing, chemical/radiation-based mutagenesis and hybridization. For transgenic lines, describe the transformation method, the number of independent lines analyzed and the generation upon which experiments were performed. For gene-edited lines, describe the editor used, the endogenous sequence targeted for editing, the targeting guide RNA sequence (if applicable) and how the editor was applied.*
Authentication	*Describe any authentication procedures for each seed stock used or novel genotype generated. Describe any experiments used to assess the effect of a mutation and, where applicable, how potential secondary effects (e.g. second site T-DNA insertions, mosiacism, off-target gene editing) were examined.*
